# Revisiting the multi-armed bandit model for the optimal design of clinical trials: benefits and drawbacks

**DOI:** 10.1186/1745-6215-14-S1-P36

**Published:** 2013-11-29

**Authors:** Sofia S Villar, Jack Bowden, James Wason

**Affiliations:** 1 MRC Biostatistics Unit, Cambridge, UK; 2 Mathematics and Statistics Department, Lancaster University, Lancaster, UK

## 

In a traditional randomised clinical trial, patients are allocated to an experimental treatment or the standard therapy arm - with equal probability - for its entire duration. However, using data from past patients to change allocation probabilities for future patients can increase the average number of patients who receive the best treatment. A drawback of doing this is that the power of the trial can be considerably reduced. The Bernoulli Multiarmed bandit problem (MABP) is an idealised model that illustrates such a conflict. For such model, Gittins & Jones (1974) provided a rule that maximises the expected patient benefit in a large trial.

Bandit models for clinical trials have been extensively studied in theory, yet the resulting schemes have been rarely used in practice. There are many reasons for this, however the power limitations to detect a significant treatment effect is a major drawback. In this presentation, we discuss allocating patients between several experimental treatments and a shared control group in a multi-arm trial, using the Gittins index. We modify the MABP design so that the shared control group is allocated separately to protect the power of the trial. The design provides considerable gains not only in efficiency over separate randomised trials but also in the average proportion of patients allocated to the best experimental treatment. This design can be advantageously extended for trials in rare diseases, where it becomes particularly relevant to optimize the choice of treatment both for patients in the trial and for those to treat after it.

